# Connexin-Dependent Transfer of cGAMP to Phagocytes Modulates Antiviral Responses

**DOI:** 10.1128/mBio.03187-19

**Published:** 2020-01-28

**Authors:** Geneviève Pépin, Dominic De Nardo, Christina L. Rootes, Tomalika R. Ullah, Sumaiah S. Al-Asmari, Katherine R. Balka, Hong-Mei Li, Kylie M. Quinn, Fiona Moghaddas, Stephane Chappaz, Benjamin T. Kile, Eric F. Morand, Seth L. Masters, Cameron R. Stewart, Bryan R. G. Williams, Michael P. Gantier

**Affiliations:** a Centre for Innate Immunity and Infectious Diseases, Hudson Institute of Medical Research, Clayton, Victoria, Australia; b Department of Molecular and Translational Science, Monash University, Clayton, Victoria, Australia; c The Walter and Eliza Hall Institute of Medical Research, Inflammation Division, Parkville, Victoria, Australia; d Department of Medical Biology, The University of Melbourne, Parkville, Victoria, Australia; e Department of Anatomy and Developmental Biology, Monash Biomedicine Discovery Institute, Monash University, Clayton, Victoria, Australia; f Australian Animal Health Laboratory, Commonwealth Scientific and Industrial Research Organisation (CSIRO) Health and Biosecurity, Geelong, Victoria, Australia; g Department of Biochemistry and Molecular Biology, Monash Biomedicine Discovery Institute, Monash University, Clayton, Victoria, Australia; h Department of Microbiology and Immunology, The Doherty Institute for Infection and Immunity, The University of Melbourne, Parkville, Victoria, Australia; i School of Clinical Sciences at Monash Health, Monash University, Clayton, Victoria, Australia; j Centre for Cancer Research, Hudson Institute of Medical Research, Clayton, Victoria, Australia; Duke University Medical Center; University of Washington; Cleveland Clinic Foundation

**Keywords:** connexins, STING, cGAMP, cGAS

## Abstract

Activation of cyclic GMP-AMP (cGAMP) synthase (cGAS) plays a critical role in antiviral responses to many DNA viruses. Sensing of cytosolic DNA by cGAS results in synthesis of the endogenous second messenger cGAMP that activates stimulator of interferon genes (STING) in infected cells. Critically, cGAMP can also propagate antiviral responses to uninfected cells through intercellular transfer, although the modalities of this transfer between epithelial and immune cells remain poorly defined. We demonstrate here that cGAMP-producing epithelial cells can transactivate STING in cocultured macrophages through direct cGAMP transfer. cGAMP transfer was reliant upon connexin expression by epithelial cells and pharmacological inhibition of connexins blunted *STING*-dependent transactivation of the macrophage compartment. Macrophage transactivation by cGAMP contributed to a positive-feedback loop amplifying antiviral responses, significantly protecting uninfected epithelial cells against viral infection. Collectively, our findings constitute the first direct evidence of a connexin-dependent cGAMP transfer to macrophages by epithelial cells, to amplify antiviral responses.

## Abstract (precis)

Recent studies suggest that extracellular cGAMP can be taken up by macrophages to engage STING through several mechanisms. Our work demonstrates that connexin-dependent communication between epithelial cells and macrophages plays a significant role in the amplification of antiviral responses mediated by cGAMP and suggests that pharmacological strategies aimed at modulating connexins may have therapeutic applications to control antiviral responses in humans.

## OBSERVATION

cGAS initiates immune responses to pathogenic and endogenous cytoplasmic DNA with long double-stranded DNA being its most potent trigger (1). Upon activation by DNA, cyclic GMP-AMP (cGAMP) synthase (cGAS) produces cGAMP that acts as a second messenger through its binding to STING (2). STING engagement by cGAMP executes a potent antiviral program through IRF3 activation and beta interferon (IFN-β) production (3). Critically, owing to its small molecular weight, cGAMP has the capacity to transfer between adjacent cells of the same tissue through the formation of gap junctions made of connexins (4). As a result, cGAMP can transactivate adjacent cells expressing STING (4, 5). In addition, cGAMP can be transferred to distal cells, including macrophages, through packaging into viral particles (6, 7).

However, whether cGAMP produced by infected cells can directly activate immune cells to bolster antiviral responses, independent of viral particle or microvesicle packaging, remains poorly defined. Recent evidence suggests that tumor-derived cGAMP can be secreted and transferred to phagocytes through an uncharacterized mechanism to instigate antitumoral immune responses (8, 9). Although engulfment of cGAMP-producing dying cells can promote STING activation in phagocytes (10), this mode of cGAMP transfer is probably not involved in the early propagation of antiviral responses and would rather be implicated in the later stages of lytic infections.

**FIG 1:**
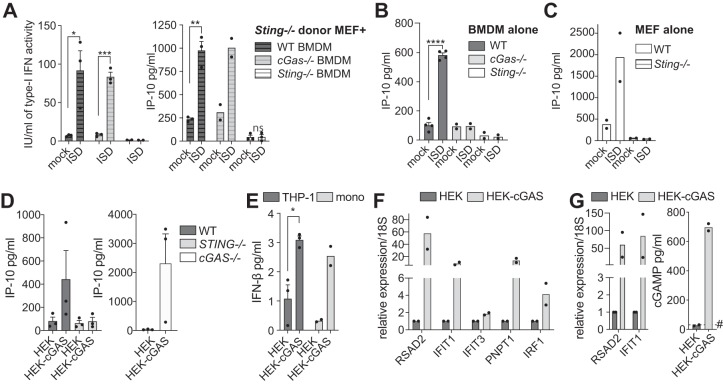
cGAS-independent, STING-dependent transactivation of macrophages by cGAMP-producing cells. (A) BMDMs (*WT*, *Sting^−/−^*, or *cGas^−/−^*) were cocultured for 18 h with *Sting^−/−^* MEFs that were previously transfected with 2 μg/ml of immunostimulatory DNA (ISD) for 2 h (and extensively washed to remove any residual ISD, as previously reported [21]). The mock condition was Lipofectamine only. Murine IP-10 (IP-10) (right panel) or type I IFN activity (left panel) levels were measured from the supernatant of the different coculture conditions. (B and C) The functionality of cGas-Sting signaling in BMDMs (B) or MEFs (C) was validated by IP-10 enzyme-linked immunosorbent assay (ELISA) following transfection of 2 μg/ml ISD for 18 h. (A to C) Data are averaged from a minimum of two independent experiments conducted in biological triplicate (± the standard errors of the mean [SEM] and unpaired *t* test results compared to mock conditions are shown). (D) IP-10 levels were measured from the supernatants of HEK or HEK-cGAS cells cocultured for 18 h with PMA-treated THP-1 monocytes (matched WT and *STING^−/−^* [D, left panel] or *cGAS^−/−^* [D, right panel]). The data shown are averaged from three independent experiments conducted in biological triplicate (± the SEM). (E) Primary human monocytes (mono) from two donors or THP-1 WT cells were cocultured for 18 h with HEK or HEK-cGAS cells and IFN-β levels measured from the supernatant. The data shown are averaged from two independent experiments conducted in biological duplicate and representative of three blood donors (primary monocytes), or are averaged from three independent experiments conducted in biological triplicate (THP-1) (± the SEM and unpaired *t* tests comparing HEK to HEK-cGAS coculture). (F and G) PMA-treated WT THP-1 cells (F) or *cGAS^−/−^* cells (G) constitutively expressing the fluorescent protein citrine (an EGFP variant) were cocultured with HEK or HEK-cGAS for 9 h before being sorted by flow cytometry based on citrine expression. Pure populations of WT THP-1-citrine cells (F) or *cGAS^−/−^-*citrine cells (G) were harvested to analyze expression of a panel of ISGs by RT-qPCR. *cGAS^−/−^* THP-1-citrine cells were also harvested to determine the level of intracellular cGAMP by specific ELISA (G, right panel). #, The limit of detection by the cGAMP ELISA. (F and G) Data shown are averaged from two independent experiments conducted in biological duplicate. (A to G) Each point represents the mean data for each independent experiment; the column represents the mean of the experiments. *, *P ≤ *0.05; **, *P ≤ *0.01; ***, *P ≤ *0.001; ****, *P ≤ *0.0001, ns, nonsignificant. Detailed materials and methods are provided in Text S1.

To confirm that viable cells can transactivate STING in phagocytes, we studied the coculture of healthy cGAMP-producing immortalized *Sting*-deficient mouse embryonic fibroblasts (MEFs) with primary bone marrow-derived macrophages (BMDMs) from wild-type (WT), *cGas*-deficient, and *Sting*-deficient mice. Coculture of DNA-transfected *Sting*-deficient MEFs resulted in *Sting*-dependent and *cGas*-independent IP-10 and type-I IFN production from BMDMs, demonstrating that cGAMP produced by the MEFs could transactivate Sting in the recipient BMDMs (Fig. 1A to C).

To transpose these findings to human cell models, we next used HEK293T cells constitutively expressing high cGAS levels (referred to as HEK-cGAS here) and producing elevated basal levels of cGAMP (4). Coculture of HEK-cGAS cells with phorbol myristate acetate (PMA)-treated THP-1 macrophages resulted in the *STING*-dependent and *cGAS*-independent production of IP-10 by the recipient THP-1 cells (Fig. 1D and see Fig. S1A in the supplemental material), which was absent in cocultures with parental HEK cells (lacking cGAS/cGAMP). Coculture of HEK-cGAS cells with WT THP-1 cells or purified primary human monocytes also increased IFN-β secretion compared to the coculture with HEK cells (Fig. 1E). Quantitative reverse transcription-PCR (RT-qPCR) analyses of fluorescent THP-1 (WT or *cGAS*-deficient) sorted by flow cytometry after coculture with HEK-cGAS cells confirmed the induction of an antiviral gene signature (with RSAD2, IFIT1, IFIT3, IRF1, and PNPT1) in the macrophages (Fig. 1F and G). Critically, cytosolic cGAMP levels were strongly increased in sorted *cGAS^−/−^* THP-1 cells after coculture with the HEK-cGAS cells, while being undetectable in control *cGAS^−/−^* THP-1 cells cocultured with WT HEK cells (Fig. 1G). Collectively, these results firmly establish the capacity of cGAMP to be transferred from viable epithelial cells to macrophages and monocytes.

10.1128/mBio.03187-19.1 FIG S1 Control experiments. (A) THP-1 *STING^–/–^* cells do not respond to ISD stimulation. IP-10 was measured from the supernatant of THP-1 WT and *STING^–/–^* cells following overnight ISD transfection using 2 μg/ml of ISD. Data shown represent the average of two different THP-1 CRISPR^−/−^ clones and matched control cells, conducted in biological triplicate. (B) WT THP-1 cells pretreated or not with PMA for 2 h, were rinsed and transfected overnight with Lipofectamine only (mock), ISD, or cGAMP, and IP-10 levels measured by ELISA. The data shown are averaged from a minimum of two independent experiments conducted in biological triplicate (± the SEM). (C) CBX treatment did not impact THP-1 cells response to cGAMP upon transfection overnight. IP-10 was measured from the supernatant of primary monocytes transfected with 1 μg/ml cGAMP using Lipofectamine in the presence or absence of CBX at 100 μM. The data shown represent the average of two independent experiments (in two blood donors) conducted in biological duplicate. (A to C) Each point represents the mean data for each independent experiment, the column representing the mean of the experiments. Download FIG S1, EPS file, 1.1 MB. Copyright © 2020 Pépin et al. 2020 Pépin et al. This content is distributed under the terms of the Creative Commons Attribution 4.0 International license.

**FIG 2:**
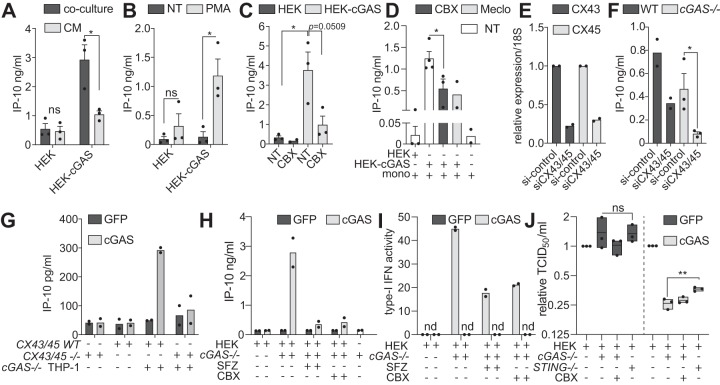
Transfer of cGAMP to macrophages is connexin-dependent and propagates antiviral responses. (A) IP-10 was measured from the supernatants of PMA-treated THP-1 cells cultured for 18 h with HEK or HEK-cGAS cells or alone and in the presence of conditioned medium (CM) from HEK or HEK-cGAS cells. Data shown are averaged from three independent experiments conducted in biological triplicate (± the SEM and unpaired *t* tests comparing coculture with CM conditions). (B) WT THP-1 cells pretreated or not with PMA for 2 h were rinsed and cocultured overnight with HEK or HEK-cGAS cells, and the IP-10 levels were measured by ELISA. The data shown are averaged from three independent experiments conducted in biological triplicate (± the SEM and unpaired *t* tests comparing NT with PMA conditions). PMA-treated WT THP-1 cells (C) or human blood-derived monocytes (mono) (D) were cocultured overnight with HEK or HEK-cGAS cells alone, in the presence of 100 μM carbonoxolone (CBX) or 100 μM meclofenamate (Meclo) to inhibit connexins. (C and D) Data are averaged from two independent experiments conducted in biological duplicate (Meclo) or three (CBX) independent experiments conducted in biological triplicate (± the SEM and unpaired *t* test results). (E) *CX43* and *CX45* mRNA levels upon cotransfection of targeting (or control) siRNAs for 24 h were measured by RT-qPCR relative to 18S. Data shown are averaged from two independent experiments conducted in biological duplicate. (F) IP-10 levels were measured by ELISA in supernatants from overnight culture of PMA-treated WT or *cGAS^−/−^* THP-1 cells with HEK-cGAS cells previously transfected with siRNAs targeting CX43 and CX45. Data shown are averaged from at least two independent experiments conducted in biological triplicate (± the SEM and unpaired *t* tests compared to the si-control). (G) HEK-*CX43/45^WT^* (*CX43/45 WT*) or HEK-*CX43/45^−/−^* (*CX43/45^–/–^*) cells were transfected with a plasmid encoding cGAS-GFP or GFP (used as control) for 1 h prior to their coculture with *cGAS^−/−^* THP-1 cells (THP-1) for 18 h. Data shown are averaged from two independent experiments conducted in biological triplicate. (H, I) HEK-Blue cells were transfected for 24 h prior washing and overnight coculture with PMA-treated THP-1 *cGAS^–/–^* (see Fig. S2A). CBX (100 μM) or SFZ (0.5 mM) was added at the time of coculture where indicated. Coculture supernatants were analyzed for IP-10 production (H) and SEAP (reflective of IFN activation of the HEK-Blue) (I). (H and I) Data shown are averaged from two independent experiments conducted in biological triplicate (I). (J) Cells treated as for panels H and I were infected for 24 h with influenza A virus (IAV) (strain A/WSN/1933[H1N1]) (MOI of 5). Then, 10-fold dilutions of supernatants were made in PBS and added to a 96-well tissue culture plate containing Vero cells in growth medium. The data shown are averaged from three independent experiments conducted in biological triplicate, reported to the mean of condition HEK-Blue expressing GFP or cGAS (± the SEM and unpaired *t* tests shown). (A to J) Each point represents the mean data for each independent experiment, the column representing the mean of the experiments.*, *P ≤ *0.05; **, *P ≤ *0.01; ns, not significant; nd, not detected. Detailed materials and methods are provided in Text S1.

Since cGAMP may be secreted by cancer cells (9) or packaged in microvesicles (6), we tested whether our observations of macrophage transactivation could be seen with conditioned medium (CM) from HEK-cGAS cells. Comparison of the activation level of THP-1 cells cocultured with HEK-cGAS to that of THP-1 cells incubated with the CM of HEK-cGAS cells demonstrated that CM alone mildly induced IP-10 but that IP-10 levels were 3-fold higher in the coculture condition (Fig. 2A): this points to a critical role for direct cellular interactions in transactivation.

10.1128/mBio.03187-19.2 FIG S2 HEK-Blue and THP-1 coculture model. HEK-Blue cells were transfected with plasmids encoding cGAS-GFP or GFP for 24 h, prior to an extensive wash and overnight coculture with PMA-treated THP-1. cGAMP produced by cGAS-GFP expressing HEK-Blue cells was transferred to THP-1 in a connexin dependent manner (inhibited by CBX), to result in IP-10 and IFN-β production by the THP-1 compartment, through STING activation. IFN-β production acted in a paracrine manner on HEK-Blue cells as measured by the ISG-SEAP reporter expression. Finally, the cells were infected with IAV for 24 h prior to viral titer assay of the virus produced in the supernatants. Download FIG S2, PDF file, 0.2 MB. Copyright © 2020 Pépin et al. 2020 Pépin et al. This content is distributed under the terms of the Creative Commons Attribution 4.0 International license.

When setting up our initial THP-1/HEK cell coculture experiments, we noticed that naive THP-1 cells did not significantly increase IP-10 production upon coculture with HEK-cGAS cells, although they produced IP-10 upon cGAS/STING engagement (Fig. 2B; Fig. S1B). Mindful of the previous report that PMA treatment of THP-1 cells increased their gap junction activity with epithelial cells (11), we speculated that gap junctions could be involved in macrophage transactivation by cGAMP-producing cells. To test this, PMA-treated THP-1 cells were cocultured with HEK-cGAS cells in the presence of carbenoxolone (CBX) (Fig. 2C), which broadly inhibits gap junction-forming connexins and cGAMP transfer between HEK cells (4). CBX robustly reduced IP-10 production in THP-1 and primary monocyte cocultures with HEK-cGAS cells (Fig. 2C and D). Meclofenamate (Meclo), another chemical gap junction inhibitor (5), mirrored the effect of CBX (Fig. 2D). Importantly, at the concentration used, CBX did not impact the IP-10 response of primary monocytes to transfected cGAMP (Fig. S1C).

Critically, downregulation of connexin 43 (CX43) and connexin 45 (CX45) by small interfering RNA transfection in HEK-cGAS cells (reducing their target mRNA by >70%; Fig. 2E), impaired IP-10 production by cocultured WT or *cGAS^−/−^* THP-1 cells (Fig. 2F). Further, HEK cells deficient in CX43 and CX45 (HEK-*CX43/45^−/−^*) and overexpressing cGAS-GFP failed to transactivate cocultured *cGAS^−/−^* THP-1, unlike their WT counterpart (*CX43/45^WT^*) (Fig. 2G). Together, our results directly implicate connexins in cGAMP transfer from cGAMP-producing cells to phagocytes.

To address the physiological relevance of cGAMP transfer to macrophages described here and its contribution to antiviral responses in uninfected tissues, we established a coculture model between *cGAS^−/−^* or *STING^−/−^* THP-1 cells and HEK-Blue cells (expressing a functional IFN-signaling axis and a secreted embryonic alkaline phosphatase [SEAP] reporter under the control of an IFN-stimulated gene [ISG] promoter; Fig. S2). HEK-Blue cells were transiently transfected with vectors encoding cGAS-GFP or green fluorescent protein (GFP), prior to overnight coculture with the THP-1 cells. In agreement with our previous findings, cGAS-GFP overexpression (but not GFP alone) resulted in the potentiation of IP-10 production in the cocultures with *cGAS^−/−^* THP-1 cells, which was blunted by CBX treatment (Fig. 2H). We also treated the coculture with sulfasalazine (SFZ) to define the putative engagement of SLC19A1, recently reported to be involved in cGAMP import in THP-1 cells (12). Surprisingly, SFZ blunted IP-10 production to a similar extent as CBX (Fig. 2H), suggesting that SLC19A1 may be involved in the import of cGAMP into the THP-1 compartment. Critically, analysis of the ISG-SEAP reporter confirmed that the THP-1 transactivation fed back into an increased ISG response in the HEK-Blue cells, involving connexins and SLC19A1 (as seen with the 50% reduction of IFN activity with CBX and SFZ treatments) (Fig. 2I).

Accordingly, this type-I IFN response in HEK-Blue cells significantly contributed to enhanced protection against influenza A virus (AIV) (strain A/WSN/1933[H1N1]) infection, which was entirely dependent on cGAS overexpression in the HEK-Blue cells, and significantly reduced when *STING* was absent in the THP-1 compartment (Fig. 2J). However, CBX failed to inhibit the antiviral effect of the coculture, which we attribute to the >50% IFN activity present in CBX-treated cocultures, possibly relating to other means of cGAMP transfer (e.g., phagocytosis of cGAMP-expressing cells [10], microvesicles [6], direct endocytosis [13], or through the importer SLC19A1 [12, 14]). Although surprising, the residual antiviral effect in cocultures of cGAS-expressing HEK-Blue cells with *STING-*deficient THP-1 may relate to STING-independent effects of cGAMP, for instance reported on the inflammasome (15). Altogether, these findings directly support the capacity of cGAMP-transfer to phagocytes to contribute to the propagation of antiviral responses to uninfected cells, which if unchecked could directly contribute to cytokine storm during infection, such as through inflammasome activation (15).

Several reports have previously suggested that epithelial cells can form gap junctions with phagocytes (11, 16, 17). Our findings establish the possible direct transfer of cGAMP and its antiviral effects from viable cells to phagocytes, involving a connexin-dependent intercellular communication. It is noteworthy that in addition to the model systems of cGAMP-producing cells used here, we were also able to demonstrate the connexin-dependent transactivation of STING in THP-1 by senescent fibroblast-like synoviocytes (FLS) physiologically engaging cGAS (Fig. S3) (18). These experiments indicate that cGAMP produced by aging FLS and transferred to joint macrophages by connexins may directly contribute to the chronic inflammation seen in joints, underlying diseases such as osteoarthritis or rheumatoid arthritis (19).

10.1128/mBio.03187-19.3 FIG S3 Senescent joint fibroblasts transactivate STING in cocultured macrophages, in a connexin-dependent manner. (A) FLS have a functional response to cytosolic DNA. IP-10 levels were measured by ELISA in supernatants of primary fibroblast-like synoviocytes (FLS) transfected with 2 μg/ml ISD or Lipofectamine only (mock) for 18 h. Data shown are averaged from FLS from three different donors in conducted biological triplicate (± the SEM and unpaired *t*-test shown). (B) Representative image of senescent FLS obtained after β-galactosidase staining (the later is used as a marker of senescence). (C) Senescent FLS were cocultured overnight with WT THP-1 cells in the presence or absence of 100 μM CBX. Data shown are averaged from FLS from four different donors in biological triplicate (± the SEM and unpaired *t* tests shown). (D) IP-10 levels were measured by ELISA from the supernatants of FLS from two different donors cocultured overnight with *STING^–/–^* THP-1 cells or their isogenic cell control. The data shown represent FLS from each patient separately, conducted in biological triplicates. (A, C, and D) Each point represents the mean data for each independent experiment, the column representing the mean of the experiments. **, *P ≤ *0.01; ***, *P ≤ *0.001. Download FIG S3, PDF file, 0.1 MB. Copyright © 2020 Pépin et al. 2020 Pépin et al. This content is distributed under the terms of the Creative Commons Attribution 4.0 International license.

Since we also observed potentiation of macrophages cultured with the conditioned medium of cGAMP producing cells, we propose that connexin-dependent transfer of cGAMP represents one of several modes of phagocyte activation, with others recently described, including engulfment of dying cells producing cGAMP (10), extracellular microvesicles (6), and the internalization of extracellular cGAMP through endocytosis (13), or through the importer SLC19A1 (12, 14). We note that while directly implicating connexins in cGAMP-producing epithelial cells in our experiments, we have not formally established that connexins operated in the phagocytic compartment to form a bona fide gap junction, and our finding that SFZ decreased transactivation to the same extent as CBX suggests a potential interplay of both mechanisms. Whether these mechanisms overlap to potentiate host antiviral responses remains to be defined in further studies. Importantly, while this manuscript was in revision, Schadt et al. showed that *cGas*-expressing mouse cancer cells (CT26) could transactivate Sting in cocultured dendritic cells, in a CX43-dependent manner (20). This independent study reinforces our claim that connexins operate a critical role in cGAMP transfer to phagocytes and collectively suggests that pharmacological inhibition of cGAMP transfer may help in curbing toxic inflammation, for instance generated during certain viral infections.

10.1128/mBio.03187-19.4 TEXT S1 Experimental details of the reagents and methods used. Download Text S1, DOCX file, 0.05 MB. Copyright © 2020 Pépin et al. 2020 Pépin et al. This content is distributed under the terms of the Creative Commons Attribution 4.0 International license.
