# Supplementary figures and images for: Connexin-Dependent Transfer of cGAMP to Phagocytes Modulates Antiviral Responses
Source: mBio. 2020 Jan 28;11(1):e03187-19. doi: 10.1128/mBio.03187-19 (PMC6989113; doi:10.1128/mBio.03187-19)

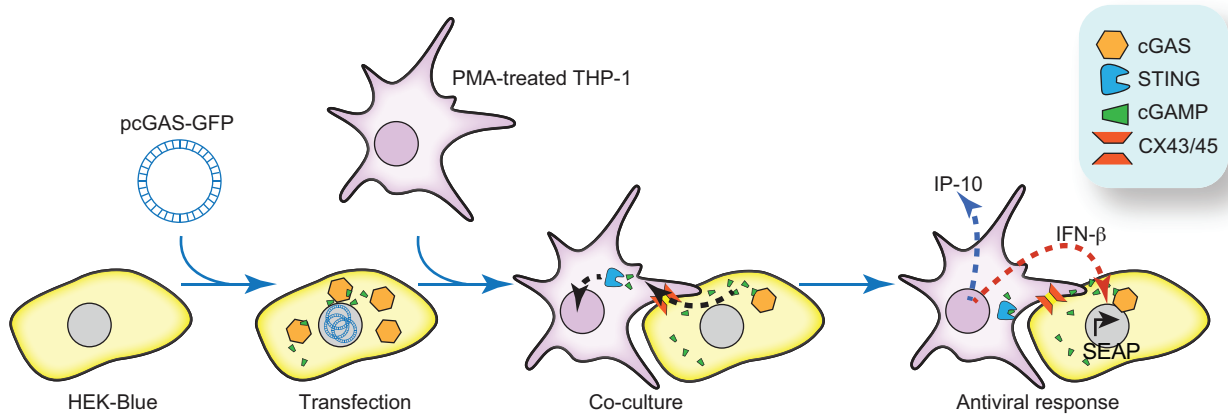

**Supplemental Figure S2**

Supplement: FIG S2 [file mBio.03187-19-sf002.pdf]

**A**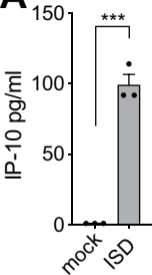**B**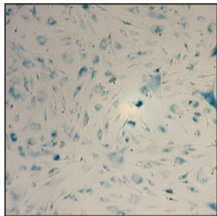**C**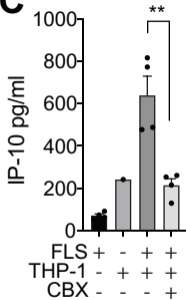**D**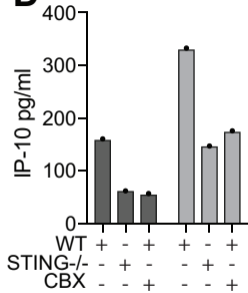

**Supplemental Figure S3**

Supplement: FIG S3 [file mBio.03187-19-sf003.pdf]
