# Supplementary material for: Connexin-Dependent Transfer of cGAMP to Phagocytes Modulates Antiviral Responses
Source: mBio. 2020 Jan 28;11(1):e03187-19. doi: 10.1128/mBio.03187-19 (PMC6989113; doi:10.1128/mBio.03187-19)
Supplement: TEXT S1 [file mBio.03187-19-s0001.docx]

**Supplemental methods**

**Ethics statement.** The use of human tissues and experimental procedures was approved by the Monash Medical Centre Ethics Committee (references MMCA/2008/26/BC and MMCA 2007/07) and the Human Research Ethics Committee. Primary fibroblast-like synoviocytes (FLS) were obtained from surgical specimens of synovial tissue. The use of mouse tissues was approved by the Monash University Animal Ethics Committee (MARP/2016/156) and (MARP/2018/067). C57BL/6 wild-type mice, *cGas*-deficient ***(1)*** mice and *Sting*-deficient mice ***(2)*** were maintained in specific-pathogen-free conditions at the Monash University Animal Research Laboratory Facility.

**Cell culture.** For bone marrow derived macrophages (BMDMs), bone marrow cells were isolated from the femurs of the mice and differentiated in 20 % L929-cell-conditioned medium for six days at 37°C in a 5% CO_2_ atmosphere, as described previously ***(3)***. Primary *Sting*-deficient and matched wild type (WT) control MEFs ***(4)***, were SV40T immortalised as previously described ***(5,6)***. HEK-cGAS^high^ cells and control cells expressing high levels of murine cGAS, HEK CX43/45^-/-^ cells and matched CX43/45^+/+^ (originally reported as HEK-Sting CX43/45^-/-^ or CX43/45^+/+^ as they stably express the murine Sting fused to an N-terminal mCherry-tag) were all previously described ***(7)***. LL171 reporter cells (L929 cells expressing an IFN stimulated response element (ISRE)-Luciferase) were previously described ***(7)***. HEK-Blue™ IFN-α/β Cells expressing a constitutive IFN signaling pathway (ISGF3) and an inducible secreted embryonic alkaline phosphatase (SEAP) reporter gene under the control of the IFN-α/β inducible ISG54 promoter were from Invivogen. MEFs, LL171, Vero cells, LentiX^TM^ 293T cells (Clontech), and HEK-Blue™ were grown in Dulbecco’s modified Eagle’s medium (Life Technologies) supplemented with 10 % heat inactivated foetal bovine serum (Life Technologies) and 1× antibiotic/antimycotic (Life Technologies) (referred to as complete DMEM). Human monocytes were prepared from blood donors. Briefly, 10 ml of human blood was used to enrich monocytes using RosetteSep™ Human Monocyte Enrichment Cocktail (Stemcell Technologies #15028). THP-1 cGAS^-/-^ cells were previously described ***(8)***, and were generated by CRISPR-Cas9 editing technology. THP-1 STING^-/-^ cells, that do not respond to DNA stimulation (Supplemental Figure S1A), and matched controls were prepared using CRISPR/Cas9 editing following a previously described protocol ***(9)***, with the following sgRNA guides (two cell lines were generated): 5’-tcccAGAGCACACTCTCCGGTACC-3’ or tcccAAGGGCGGGCCGACCGCATT. THP-1, monocytes and FLS were grown in RPMI 1640 plus L-glutamine medium (Life Technologies) complemented with 1x antibiotic/antimycotic and 10% heat inactivated foetal bovine serum (referred to as complete RPMI). For co-culture studies, THP-1 cells were pre-treated with Phorbol-12-myristate-13-acetate (PMA, Merck #524400) for 2 h at 20 ng/ml. When needed, cells were treated with Carbenoxolone (CBX, Sigma #C4790 - resuspended in H_2_O), Meclofenamate (Meclo, Sigma #M4531 - resuspended in DMSO), or sulfasalazine (SFZ, Sigma #S0883 - resuspended in DMSO). Transfection of IFN stimulatory DNA (ISD) was performed using either a 40 long dsDNA for mouse cell lines (TAC AGA TCT ACT AGT GAT CTA TGA CTG ATC TGT ACA TGA TCT ACA – annealed with complementary sequence) or 70 long dsDNA for human cell lines (CCA TCA GAA AGA GGT TTA ATA TTT TTG TGA GAC CAT CGA AGA GAG AAA GAG ATA AAA CTT TTT TAC GAC T -annealed with complementary sequence) at a concentration of 2 μg/ml at a ratio of 1 μg:1 μl with Lipofectamine 2000. cGAMP (Sigma, #SML1229) was transfected at 1 μg/ml using Lipofectamine 2000 at a ratio of 1 μg:1 μl. Mock conditions were with Lipofectamine 2000 only.

**Co-culture studies.** HEK-cGAS or HEK-WT cells were co-cultured overnight with monocytes (primary or THP-1) at a ratio of 50,000 HEK: 50,000 monocytes respectively in a well of a 96 well-plate. HEK-Sting cells (CX43/45^-/-^ or CX43/45^+/+^) were transfected with murine cGAS-GFP plasmid ***(10)*** (a kind gift from V. Hornung), prior co-culture with THP-1 cells. Briefly, 0.2 μg of DNA was reverse-transfected in 100,000 HEK-Sting cells using Lipofectamine 2000 in a 96 well plate. After 2 h incubation, the cells were co-cultured with 50,000 PMA-treated THP-1 cGAS^-/-^ cells and incubated for 18 h prior to supernatant analysis by ELISA (Figure 2G). For Figures 2H, 2I and 2J, 1.2 μg of DNA (pcGAS-GFP or pEGFP-N2 [Clontech]) was reverse-transfected with Lipofectamine 2000 in 500,000 HEK-Blue™ in a well of a 6 well plate. After 18-24 h incubation, the cells were collected by vigorous pipetting, spun down, washed with 10 ml DMEM, spun down, resuspended in 1.3 ml DMEM complete, prior to being plated at ~50,000 cells/well by 100 μl in 12 wells of a 96 well plate. 50,000 THP-1 treated for 2 h with 20 ng/ml of PMA were directly added and the cells were co-cultured overnight. The next day (cGAS-GFP and EGFP expression was validated by inverted microscopy in >50% of the HEK-Blue™ cells), supernatants were analyzed for SEAP or IP-10 production, or cells were infected with virus. For SEAP quantification, 25 μl of supernatant was diluted with 135 μl of QUANTI-blue (Invivogen), and incubated at 37C for 1-2h, prior reading at 650 nm using a Fluostar OPTIMA (BMG Labtech) plate reader. Recombinant IFN-α2 standards (Roferon, Roche) (series diluted from 1000 IU/ml to 7.8125 IU/ml) were used on HEK-Blue™cells treated overnight in parallel in each experiment, to determine the IFN activity in the co-cultures based on QUANTI-blue absorbance. For further infected with IAV (strain A/WSN/1933[H1N1]) at MOI of 5 for 24 h. 10-fold dilutions of IAV-containing supernatants were made in PBS and added to a 96-well tissue culture plate containing Vero cells in growth medium. Plates were incubated for 5 days at 37°C, 5% CO_2_ and scored for cytopathic effect. The infectious titer was calculated by the method of Hawkes ***(11)***. FLS were co-cultured at a ratio of 20,000 FLS for 50,000 monocytes. A β-Galactosidase staining assay was performed on FLS using the Senescence β-Galactosidase Staining kit (New England Biolabs, #9860). Briefly, FLS were stained in parallel to the co-culture experiments. Cells were washed with PBS, fixed and stained overnight with X-Gal according to the manufacturer’s protocol.

**Citrine-overexpression.** Stable THP-1 cells expressing Citrine from pRP-Citrine retroviral constructs were generated as follows. Briefly, 60% confluent LentiX^TM^ 293T cells were transfected with 10 μg of pRP-Citrine (gift from Eicke Latz, University of Bonn), 1 μg of VSG-g and 10 μg of gag-pol constructs, with BES-adapted method of calcium phosphate. Media was replaced 24 h after cell transfection. Two days after transfection, viral-containing supernatant was filtered at 0.45 μm and supplemented with 8 μg/ml of polybrene (Sigma, #107689). 1.5 ml of viral supernatant was added to 1 ml of cell suspension containing 2x10^6^ THP-1 cells for 3 hours. Then, the virus-containing media was replaced with fresh media and the cells were incubated for another 48 h prior to the addition of puromycin at 2 μg/ml in fresh complete RPMI medium. Cells were passaged every three days (with complete RPMI containing puromycin) until the control cells (without transduction) died (around 2-3 passages). Citrine expression in THP-1 cells was confirmed by flow cytometry analysis.

**Cytokines analysis:** For the murine type-I IFN activity assay, LL171 reporter cells were seeded the day before at 20,000 cells per well in a 96 well plate in 100 μL of complete media. 100 μL of neat supernatant from the MEF and BMDM cultures was added to the LL171 cells as described previously (26). Luciferase activity was analysed 6 h later. Serial dilution of recombinant murine IFN-β (gift from N.A. de Weerd, A. Matthews and P.J. Hertzog, Hudson Institute) was used to prepare a standard curve. Murine IP-10 production in supernatant of MEF cells, BMDM or co-cultures of MEF cells and BMDM after ISD transfection was quantified using Mouse CXCL10/IP-10/CRG-2 Duo Set ELISA (R&D systems, #Dy466) according to the manufacturer’s protocol. Similarly, human IP-10 production and IFN-β were measured using 100 μL of supernatants from the different co-cultures and were quantified using IP-10 ELISA kit (BD Biosciences, # 555157) or IFN-β (PBL assay science, #41410) respectively, according to the manufacturer’s protocol. Intracellular cGAMP was measured by lysing cells 300,000 cells in 200 μL of M-PER lysis buffer (Thermo Fisher, #78501) according to the manufacturer’s protocol. Cleared lysates (50 μL per sample) were used to quantitate cGAMP using 2'3'-cGAMP ELISA kit (Cayman chemical, #501700).

**Reverse transcription quantitative real-time PCR (RT-qPCR).** Total RNA was purified from cells using the ISOLATE II RNA Mini Kit (Bioline). For mRNA quantification, cDNA was synthesized from isolated RNA using the High-Capacity cDNA Archive kit (Life Technologies) according to the manufacturer’s instructions. RT-qPCR was carried out with the Power SYBR Green Master Mix (Applied Biosystems) on the HT7900 RT-PCR system (Life Technologies). Each PCR was carried out in technical duplicate and human 18S was used as reference genes. Each amplicon was used to generate a standard curve for the quantification of gene expression (used in each run). Melting curves were used in each run to confirm specificity of amplification. The primers used were the following: Human RSAD2: hRSAD2-RT-FWD TGGTGAGGTTCTGCAAAGTAG; hRSAD2-RT-REV GTCACAGGAGATAGCGAGAATG; hIFIT1: hIFIT1-FWD TCACCAGATAGGGCTTTGCT; hIFIT1-REV CACCTCAAATGTGGGCTTTT; hIFIT3: hIFIT3-FWD CATAAAAGCACAGACCTAACAGC; hIFIT3-REV CAGGGAATTCTTGGTGACCTC; hIRF1: hIRF1-FWD CCCTGCCAGATATCGAGGAG, hIRF1-REV CTCGCACAGCTGAGCTGC; hPNPT1: hPNPT1-FWD TGCAGTAATGGTCACAGCGG; hPNTP1-REV TGTAGTCAACCACCAAAGGCA hCX43: hCX43-FWD TTTAAGCAAAAGAGTGGTGCCC; hCX43-REV AGACTGCTCATC TCCCCAGG; hCX45: hCX45 FWD GGAAGACCTCCGTCTGGATT; hCX45-REV AGTCCC CTGAGCTTGGATCAT; h18S: h18S-FWD CGGCTACCACATCCAAGGAA; h18S-REV GCTGGAATTACCGCGGCT.

**RNA interference.** 50,000 HEK-cGAS per well (96-well plate) were reverse-transfected with 7 nM of each siRNA (2.25 μl RNAiMax / 2.1 μl of each siRNA at 2 μM for 3 wells with 200 μl final volume) for 24 h, before being co-cultured with 50,000 PMA-treated THP-1 cells (WT or cGAS^-/-^). All siRNAs were synthesized as pre-annealed Dicer-substrates by Integrated DNA Technologies (r = RNA base and Uppercase = DNA base). Sequences used were: human siCX43-S rGrUrA rCrArA rGrCrA rGrArU rArCrA rGrUrA rUrArA rArCT C; siCX43-AS rGrArG rUrUrU rArUrA rCrUrG rUrArU rCrUrG rCrUrU rGrUrA rCrCrA; siCX45-S rGrArA rUrCrC rArUrC rUrArU rUrArC rGrArU rGrArG rCrAA A; siCX45-AS rUrUrU rGrCrU rCrArU rCrGrU rArArU rArGrA rUrGrG rArUrU rCrUrC.

**Statistical analyses.** Statistical analyses were carried out using Prism 8 (GraphPad Software Inc.). Two-tailed unpaired *t*- tests were used to compare pairs of conditions, Symbols used: * *P*≤0.05, ** *P*≤0.01, *** *P*≤0.001, **** *P*≤0.0001.

**References**

1. Schoggins JW, MacDuff DA, Imanaka N, Gainey MD, Shrestha B, Eitson JL, Mar KB, Richardson RB, Ratushny AV, Litvak V, Dabelic R, Manicassamy B, Aitchison JD, Aderem A, Elliott RM, Garcia-Sastre A, Racaniello V, Snijder EJ, Yokoyama WM, Diamond MS, Virgin HW, Rice CM (2014) Pan-viral specificity of IFN-induced genes reveals new roles for cGAS in innate immunity. Nature 505 (7485):691-695. doi:10.1038/nature12862

2. Jin L, Hill KK, Filak H, Mogan J, Knowles H, Zhang B, Perraud AL, Cambier JC, Lenz LL (2011) MPYS is required for IFN response factor 3 activation and type I IFN production in the response of cultured phagocytes to bacterial second messengers cyclic-di-AMP and cyclic-di-GMP. J Immunol 187 (5):2595-2601. doi:10.4049/jimmunol.1100088

3. Ferrand J, Gantier MP (2016) Assessing the Inhibitory Activity of Oligonucleotides on TLR7 Sensing. Methods Mol Biol 1390:79-90. doi:10.1007/978-1-4939-3335-8_5

4. Pepin G, Ferrand J, Honing K, Jayasekara WS, Cain JE, Behlke MA, Gough DJ, Williams BRG, Hornung V, Gantier MP (2016) Cre-dependent DNA recombination activates a STING-dependent innate immune response. Nucleic Acids Res 44 (11):5356-5364. doi:10.1093/nar/gkw405

5. Gantier MP, McCoy CE, Rusinova I, Saulep D, Wang D, Xu D, Irving AT, Behlke MA, Hertzog PJ, Mackay F, Williams BR (2011) Analysis of microRNA turnover in mammalian cells following Dicer1 ablation. Nucleic Acids Res 39 (13):5692-5703. doi:10.1093/nar/gkr148

6. Pepin G, Nejad C, Thomas BJ, Ferrand J, McArthur K, Bardin PG, Williams BR, Gantier MP (2017) Activation of cGAS-dependent antiviral responses by DNA intercalating agents. Nucleic Acids Res 45 (1):198-205. doi:10.1093/nar/gkw878

7. Ablasser A, Schmid-Burgk JL, Hemmerling I, Horvath GL, Schmidt T, Latz E, Hornung V (2013) Cell intrinsic immunity spreads to bystander cells via the intercellular transfer of cGAMP. Nature 503 (7477):530-534. doi:10.1038/nature12640

8. Mankan AK, Schmidt T, Chauhan D, Goldeck M, Honing K, Gaidt M, Kubarenko AV, Andreeva L, Hopfner KP, Hornung V (2014) Cytosolic RNA:DNA hybrids activate the cGAS-STING axis. EMBO J 33 (24):2937-2946. doi:10.15252/embj.201488726

9. Baker PJ, Masters SL (2018) Generation of Genetic Knockouts in Myeloid Cell Lines Using a Lentiviral CRISPR/Cas9 System. Methods Mol Biol 1714:41-55. doi:10.1007/978-1-4939-7519-8_3

10. Civril F, Deimling T, de Oliveira Mann CC, Ablasser A, Moldt M, Witte G, Hornung V, Hopfner KP (2013) Structural mechanism of cytosolic DNA sensing by cGAS. Nature 498 (7454):332-337. doi:10.1038/nature12305

11. Hawkes RA (1979) General principles underlying laboratory diagnosis of viral infections. Diagnostic procedures for viral, rickettsial and chlamydial infections 3
